# Intramedullary Screw Fixation of a Proximal Fifth Metatarsal Stress Fracture in an Elite Athlete: A Case Report

**DOI:** 10.1055/s-0037-1599833

**Published:** 2017-03-17

**Authors:** Steffen Sauer

**Affiliations:** 1 Department of Orthopaedic Surgery and Sports Medicine, Aarhus University Hospital, Aarhus, Denmark

**Keywords:** intramedullary screw fixation, proximal fifth metatarsal fracture, pes cavus, hindfoot varus

## Abstract

Intramedullary screw fixation of proximal fifth metatarsal fractures is a simple surgical procedure, enabling early postoperative weight-bearing and subsequently rapid return to competitive sport, which is of great significance for elite athletes. The procedure is described in an elite basketball player in this article. Pes cavus and hindfoot varus alignment potentiate cyclic loading onto the fifth metatarsal and should be addressed as it may represent underestimated factors concerning fracture prognosis.

First described by Kavanaugh et al in 1978, intramedullary screw fixation is still indicated for proximal fifth metatarsal fractures. 1 The simple percutaneous technique allows stable fracture fixation and thus early postoperative weight-bearing and rapid return to preinjury level of competitive sport. 2 However, in terms of prognosis, tuberosity avulsion fractures ( Fig. 1 ; zone 1) must be distinguished from both traumatic fractures at the metaphyseal–diaphyseal junction (true Jones fracture; Fig. 1 ; zone 2) as well as proximal diaphyseal stress fractures ( Fig. 1 ; zone 3). The latter are a result of repetitive loading leading to failure of the skeletal structure and are associated with longer consolidation times and complications. 3 Pes cavus and hindfoot varus foot alignment has been identified to potentiate cyclic loading onto the fifth metatarsal, which favors the incidence of a stress fracture. What is more, diaphyseal blood supply of the fifth metatarsal is ensured solely by the nutrient artery and may therefore be compromised when a diaphyseal fracture occurs, which may subsequently impede fracture healing. 4 In spite of excellent results regarding intramedullary screw fixation for proximal fifth metatarsal fractures, screw breakage or bending has been reported and seems to be related to postoperative early weight-bearing, high patient body mass index (BMI), and the use of undersized screws. 5 6 7 An intramedullary screw fitting tightly may be of paramount importance.

**Fig. 1:**
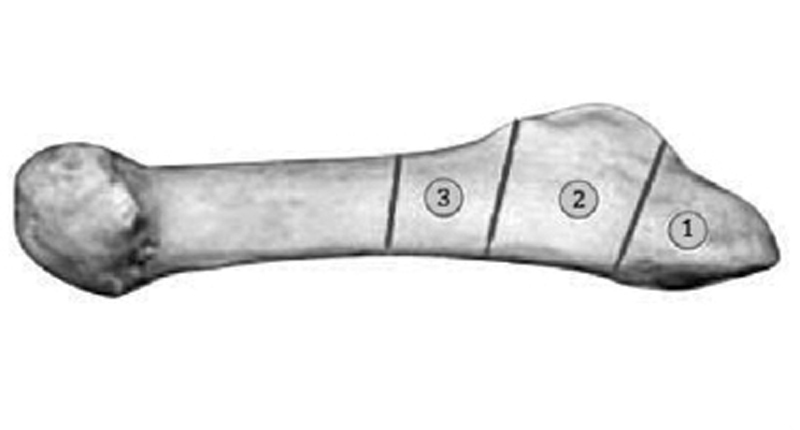
Anatomical division of the proximal aspect of the fifth metatarsal into three zones. 1, Styloid avulsion fracture (metatarsocuboid joint); 2, Jones fracture (metaphyseal–diaphyseal junction); 3, diaphyseal stress fracture.

## Case Report

**Fig. 2:**
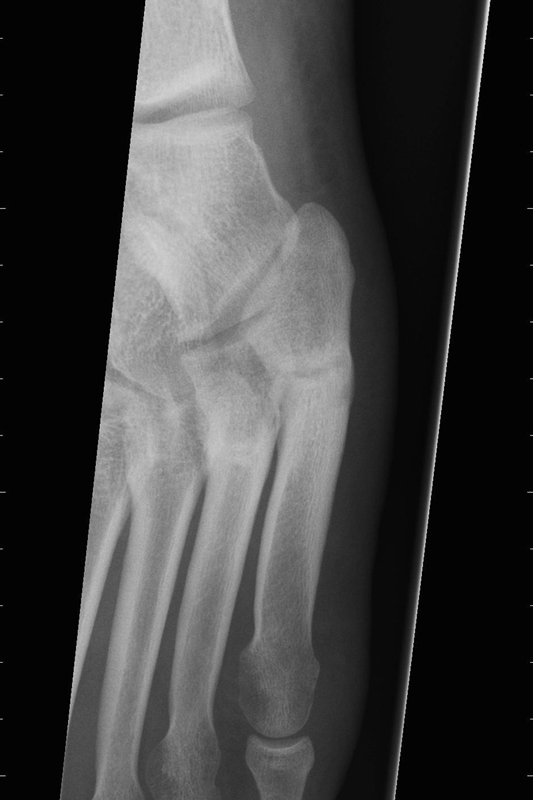
Transverse stress fracture.

**Fig. 3:**
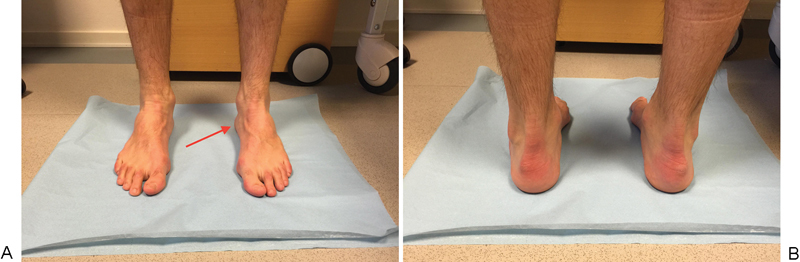
( **A** ) Peek-a-boo heel sign. ( **B** ) Hindfoot varus alignment.

A 19-year-old male professional basketball player presents with pain to the lateral border of his left foot. The symptoms commence immediately after perceiving a snap to the left foot while attempting to jump. Forced inversion or flexion of the foot during injury is denied. Radiographs confirm a transverse stress fracture of the fifth metatarsal ( Fig. 2 ). Upon clinical examination, bilateral pes cavus and hindfoot varus alignment including peek-a-boo heel sign is observed ( Fig. 3A, B ). The patient is scheduled for intramedullary screw fixation with a 4.5-mm cannulated screw.

### Surgical Technique

**Fig. 4:**
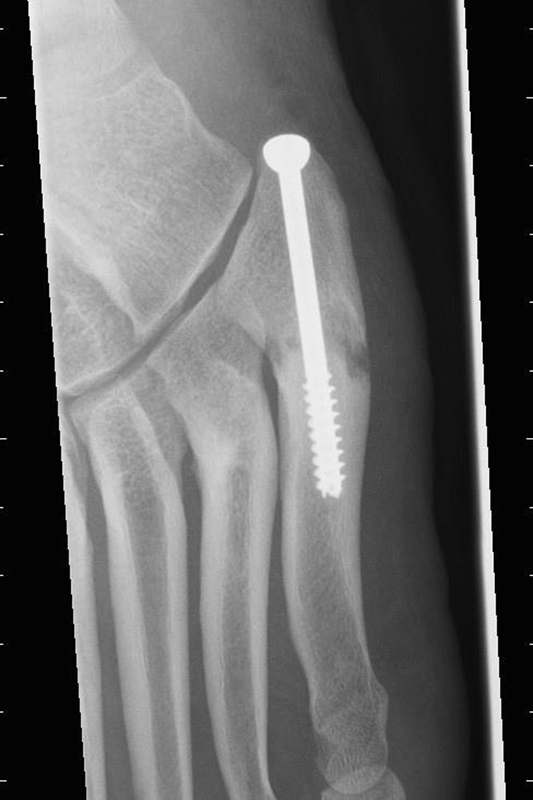
Immediately after surgery.

The surgical procedure was performed in regional anesthesia. The fracture was identified using a fluoroscan C-arm, and a small incision was made proximal to the base of the fifth metatarsal. An intramedullary guidewire was inserted into the fifth metatarsal and placed across the fracture site. The guidewire was then overdrilled protecting the soft tissue and tendon attachment with a drill sleeve. Counterpressure was placed along the longitudinal axis of the fifth metatarsal, and a correct length screw was inserted and tightened. All threads were placed distal to the fracture site. The screw head was embedded into the cortical part of the styloid process of the fifth metatarsal, avoiding screw head irritation ( Fig. 4 ). Fracture stability was ensured. The incision was closed with a single suture. The patient was equipped with a controlled ankle motion boot for 4 weeks, and nonweight-bearing for 14 days was advised.

### Follow-Up

**Fig. 5:**
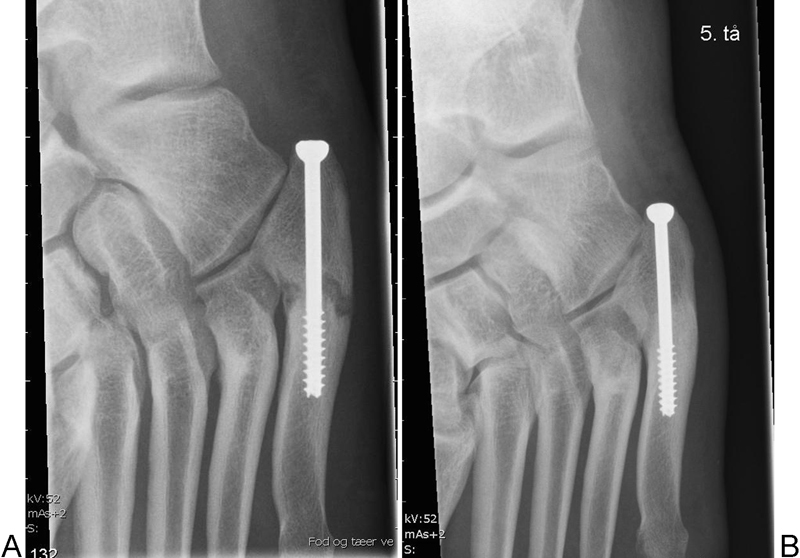
( **A** ) Five weeks after surgery. ( **B** ) Ten weeks after surgery.

The patient was assessed after 5 and 10 weeks after surgery, and radiographs of the fracture site were obtained ( Fig. 5A, B ). After 5 weeks after surgery, the patient was free of symptoms during functional activity and upon clinical examination. Return to competitive sport was permitted. After 10 weeks after surgery, radiographs demonstrated complete fracture consolidation, and the patient was free of symptoms during competitive sport and upon clinical examination. The patient was equipped with a varus unloading orthotic insert to reduce the risk of fracture recurrence. Hardware removal was not indicated.

## Discussion

Intramedullary screw fixation of proximal fifth metatarsal fractures is a relatively facile surgical procedure. Fracture fixation is ensured avoiding open reduction, which may compromise blood supply to the fifth metatarsal. Early postoperative weight-bearing and subsequently rapid return to competitive sport is enabled, which is of great significance for elite athletes. The use of a standard size screw is not advisable as it needs to be adapted to individual anatomical characteristics of the fifth metatarsal. However, an intramedullary screw fitting tightly may be of paramount importance. Although rarely in comparison to conservative treatment, nonunions, delayed unions, and refracture continue to be reported following intramedullary screw fixation and seem to be related to high patient BMI and the use of undersized screws, as well as early postoperative weight-bearing. Nonweight-bearing in a posterior splint for 2 weeks after surgery has been advised by many experts. Return to normal activity is usually achieved after 4 to 6 weeks. However, it must be based upon the absence of pain upon clinical examination and during functional activities. Hindfoot varus and pes cavus may potentiate cyclic stress on the fifth metatarsal, favoring the incidence of a stress fracture. Thus, a thorough clinical examination addressing foot alignment abnormalities is crucial as a varus unloading (lateral hindfoot and forefoot posting) orthotic insert may reduce the risk of complications such as fracture recurrence. Distinguishing acute fractures from stress fractures may be difficult and confusing. Furthermore, it is controversially discussed to what extend a precise distinction of acute fractures and stress fractures is generally indicated, as treatment protocols are identical, showing equivalent outcomes. 8 Additionally, the term *Jones fracture* is used controversially. Sir Robert Jones originally described the fracture in 1902, after self-sustaining the injury while dancing. In 1987, Lehman et al 6 proposed a classification of proximal fifth metatarsal fractures distal to the tuberosity based upon radiographic criteria, which may determine treatment strategy. Type I fractures with the absence of intramedullary sclerosis have high healing potential and may be treated conservatively. Type II fractures with widening, periosteal reaction, and/or intramedullary sclerosis represent delayed unions and may eventually heal when treated conservatively. However, surgery is preferred in active patients. Type III fractures with complete sclerosis at the fracture line represent a pseudarthrosis, and surgery is usually indicated. In 1995, Dameron and Quill proposed a classification based upon anatomical fracture location ( Fig. 1 ). A consensus regarding diagnosis, classification, pathomechanism, and treatment of proximal fifth metatarsal fractures is yet to be found. However, clinical findings such as varus hindfoot alignment and pes cavus may represent underestimated factors concerning fracture prognosis and should be addressed.
